# Insights Into Extracellular Vesicle/Exosome and miRNA Mediated Bi-Directional Communication During Porcine Pregnancy

**DOI:** 10.3389/fvets.2021.654064

**Published:** 2021-04-15

**Authors:** Mallikarjun Bidarimath, Harshavardhan Lingegowda, Jessica E. Miller, Madhuri Koti, Chandrakant Tayade

**Affiliations:** ^1^Department of Pathobiological Sciences, School of Veterinary Medicine, Louisiana State University, Baton Rouge, LA, United States; ^2^Department Biomedical and Molecular Sciences, Queen's University, Kingston, ON, Canada; ^3^Department of Obstetrics and Gynecology, Queen's University, Kingston, ON, Canada

**Keywords:** angiogenesis, cytokines, exosomes, fetal loss, immune cells, inflammation, trophoblasts

## Abstract

Spontaneous fetal loss is one of the most important challenges that commercial pig industry is still facing in North America. Research over the decade provided significant insights into some of the associated mechanisms including uterine capacity, placental efficiency, deficits in vasculature, and immune-inflammatory alterations at the maternal-fetal interface. Pigs have unique epitheliochorial placentation where maternal and fetal layers lay in opposition without any invasion. This has provided researchers opportunities to accurately tease out some of the mechanisms associated with maternal-fetal interface adaptations to the constantly evolving needs of a developing conceptus. Another unique feature of porcine pregnancy is the conceptus derived recruitment of immune cells during the window of conceptus attachment. These immune cells in turn participate in pregnancy associated vascular changes and contribute toward tolerance to the semi-allogeneic fetus. However, the precise mechanism of how maternal-fetal cells communicate during the critical times in gestation is not fully understood. Recently, it has been established that bi-directional communication between fetal trophoblasts and maternal cells/tissues is mediated by extracellular vesicles (EVs) including exosomes. These EVs are detected in a variety of tissues and body fluids and their role has been described in modulating several physiological and pathological processes including vascularization, immune-modulation, and homeostasis. Recent literature also suggests that these EVs (exosomes) carry cargo (nucleic acids, protein, and lipids) as unique signatures associated with some of the pregnancy associated pathologies. In this review, we provide overview of important mechanisms in porcine pregnancy success and failure and summarize current knowledge about the unique cargo containing biomolecules in EVs. We also discuss how EVs (including exosomes) transfer their contents into other cells and regulate important biological pathways critical for pregnancy success.

## Introduction

The pig industry around the world produces more than 100 million of pork annually and the value of U.S. and Canadian pork and pork products exports to the world reached a record $11 billion (1). Due to their high productive and reproductive efficiencies, the pigs contribute to the lower cost of pork production (2, 3). An average of 23.6 piglets per year can be produced by a single sow (4). Therefore, the litter size remains the prime contributing factor for greater yield of pork production. The litter size per gestation can be influenced by several entities such as ovulation rate, fertilization rate, and establishment of pregnancy and conceptus development and survival until the term. Pigs are highly prolific livestock species due to their certain reproductive characters such as higher ovulation rate (20−25 oocytes per cycle) (4) and fertilization rate (95%) (4–6). However, the litter size reduces to 10–13 piglets per sow, by the time they reach full term (7). The huge gap between these parameters is likely explained by a phenomenon called as spontaneous fetal loss, which occurs in two waves (8). A 20–30% of the conceptus are lost during the peri-attachment [gestation day (gd) 12–20] period (8) and an additional loss of 10–15% occurs during mid to late gestation, reviewed by (7, 9, 10). During early pregnancy, a delicate balance is absolutely critical between developing conceptus and the maternal immunomodulatory mechanisms (11). The porcine placentation is non-invasive, diffuse, epitheliochorial type, which is characterized by neither decidualization of the endometrium like in humans and mice nor invasion of fetal tissues into the endometrium, but instead both the compartments lie in close, yet firm adhesion (12, 13). Research over last decades has pointed out at several factors that are crucial to fetal development such as genetic makeup of the animal (14), nutrient intake (15), placental development and homeostasis (16), uterine capacity (14, 17), deficits in placental vasculature (18), disease outbreaks (19), immune mechanisms (20), and environment (21, 22) that specifically cause fetal loss. A variation in conceptus elongation rate and embryonic growth in early gestation especially around the peri-attachment period greatly alters the uterine environment, and thus negatively influencing the conceptus growth resulting in less developed conceptuses or even fetal demise (8). A similar variation in growth of littermates during the mid and late gestation leads to unequal space acquisition within the uterine lumen. The fetuses with comparatively higher growth rate exceed their uterine space, which pushes and compresses adjacent slow growing littermates resulting in stress/hypoxia induced conceptus arrest (8, 23, 24).

Successful porcine pregnancy is dependent on conceptus attachment and placental development, which requires a bidirectional communication between conceptus and endometrium. Previous studies have suggested many important factors that play a crucial role in pregnancy success. These factors include but not limited to conceptus-derived estrogen and growth factors (25), progesterone (26, 27), immune cells (18, 28–30), cytokines (18), chemokines (31, 32), miRNAs (29, 33), EVs including exosomes (34, 35), mRNA destabilizing factors (36), pro- and anti-angiogenic immune-related miRNAs (29, 34), and seminal fluid derived factors (37–41). However, most of these published studies only associate findings with fetal loss and a more comprehensive understanding of cause and effect relationship is required. In this review, we provide overview of important mechanisms associated with successful pregnancy or failure. We also summarize how contents from fetal and maternal derived EVs (including exosomes) contribute to pregnancy associated physiological adaptations.

## Immune Mechanisms At the Porcine Maternal-Fetal Interface

Pleiotropic glycoprotein molecules such as cytokines are secreted by a variety of immune and non-immune cells in the uterine microdomain. Cytokines regulate several pregnancy related mechanisms including inflammation (42, 43), angiogenesis (18), innate and adaptive immune responses and cell death (44). These processes particularly influence the elongation and attachment of growing conceptus, endometrial adaptation to paternally derived antigens, successful conceptus attachment and overall development. Research over the past few years has characterized the role of members of the transforming growth factor beta superfamily, interferons (especially IFN-γ and IFN-δ), and interleukins (IL-1A, IL-1B, IL-1B2, IL-6) (44–46). During gd 9–10 and gd 15–18, there is dramatic change in the endometrial expression of TNFα, tumor necrosis factor alpha-inducible protein 6 (TNFAIP6), inter-α-trypsin inhibitor heavy chains (ITIH), and IL-6 that regulate the extracellular matrix expansion. IFN-γ is typically secreted around gd12 and dramatically increases around gd16 (47, 48). In our previous studies, the fetal loss during the peri-attachment period coincided with increased IFN-γ expression, while its expression was unaltered during a second wave of fetal loss around mid-pregnancy (gd50) (49). The transient increase in IFN-γ expression between gd12 and 15 influences the immune cell recruitment and differentiation at the maternal-fetal interface (50, 51). We observed increased expression of inflammatory cytokines such as TNF-α, IL-1B, and IL-1 receptor at the conceptus attachment sites associated with arresting/dying conceptus during the peri-attachment period (49). These studies suggest that these pro-inflammatory cytokines initiated acute inflammatory response that results in the conceptus loss and resorption. Although counter argument of immune cell infiltration and their functional products, cytokines, are released to clear dying/dead conceptus cannot be fully ruled out. This adds to the complexity of cause and effect question associated with fetal loss.

Immune cell recruitment to the maternal-fetal interface in porcine pregnancy is largely driven by conceptus derived signals and set of specific chemokines and growth factors (52–54). Secreted by several immune cell types, chemokines are small signaling molecules predominantly involved in the recruitment and activation of circulating leukocytes to the sites of inflammation (55). During early pregnancy, a remodeling in the uterine microdomain happens at the sites of conceptus attachment. Therefore, the chemokines secreted by uterine epithelial cells, fibroblasts as well as resident immune cells would act to influence an increased extravasation and chemotaxis along the concentration gradient (54). However, a delicate balance is critical between a chemotaxis induced inflammatory processes and tissue homeostasis around the days of conceptus attachment and establishment of pregnancy.

Our previous research has explored these avenues by investigating the role of transcripts encoding decoy receptors, D6, duffy antigen receptor for chemokines (DARC) and chemocentryx decoy receptors (CCX-CKR). The mRNAs for decoy receptors (DARC and CCX-CKR) were dysregulated in the endometrium and chorioallantoic membrane; however, there was no difference in their expression at the protein level. A post-transcriptional and/or epigenetic modification of chemokines and their specific receptors, as well as action by proteolytic enzymes could explain these differences in expression during pregnancy (32). In another study, we investigated a distinct set of chemokines and their receptors that likely play a role in immune cell associated functions in laser microdissected endometrial lymphocytes, endometrium, and chorioallantoic membrane derived from both the arresting and healthy conceptus attachment sites. We demonstrated that lymphocytes residing in the arresting conceptus attachment sites had higher expression of *CXCR3* and *CCR5* mRNA, and there was greater expression of *CXCL10, CCL5, CCR5*, and *CXCR3* mRNA in the endometrium around peri-attachment period (gd20). Recently, Han and colleagues examined the expression of CXCL12 and CXCR4 at the maternal-conceptus interface during pregnancy in pigs. This study reported the highest expression of CXCL12 on day 15of pregnancy. Furthermore, CXCL12 protein expression was localized in endometrial epithelial cells, however, CXCR4 protein was detected in vascular endothelial cells, subepithelial stromal cells, and endometrial immune cells. CXCL12 increased the migration of cultured porcine trophectoderm cells and peripheral blood mononuclear cells as well as along with CXCR4 induced the migration of trophectoderm cells and T cells at the implantation in pigs. These experiments highlight role of CXCL12 in regulating trophoblast migration and T cell recruitment into the endometrium during implantation period in pigs (56). Recently, a study screened a broad range of chemokines (*CCL2, CCL4, CCL5, CCL8, CXCL2, CXCL8, CXCL10*, and *CXCL12*) and their receptors (CCR1, CCR2, CCR3, CCR5, CXCR2, CXCR3, and CXCR4) in luminal epithelial cells. This study suggests that CCL8 actively participates in embryo implantation and CXCL12 participates in endometrial receptivity and promotion of embryo attachment (57). Similarly, the same group screened the expression of same set of chemokines in endometrial stromal and endothelial cells and suggested that CCL2 modulates stromal cell function and CCL4 and CCL8 stimulate blood vessel development (58). These studies suggest that a further characterization of these chemokines and their specific receptors including decoy receptors may provide a better understanding of their role in immunomodulatory activity and overall function during conceptus attachment and subsequent development (31).

Previous studies in our laboratory and others have identified deficits in angiogenic stimuli that likely results in impaired vascular development at the maternal-fetal interface (18, 29, 49, 59). This is one of the important primary causes that leads to either stunted growth in surviving fetuses or fetal mortality during early stages of pregnancy. Various immune cell types have been evaluated for their crucial role during porcine pregnancy. Around the peri-attachment period (gd 12–15), the vascular remodeling of the endometrium coincides with infiltration of various innate and adaptive immune cells including natural killer cells, dendritic cells, macrophages, lymphocytes and plasma cells (60–62). The natural killer cells are generally detectable at gd 12 in pigs, which typically aggregate beneath the uterine luminal epithelium, around uterine glands, perivascular areas as well as randomly scattered throughout the uterine stroma (60, 61, 63).

It is important to note that the conceptus attachment to the endometrium is critical for the recruitment of uterine natural killer (uNK) cells in pigs as the decidualization is not induced in these species. However, in case of humans, mice and rats, where decidualization occurs, presence of conceptus is not critical for uNK cell recruitment (61, 64). T cells and uNK cell mobilization to the sites of blastocyst attachment and placentation typically noticed between gd 15–28 (65). However, their enrichment differs in various species. Compared to species with hemochorial placentation (human, mice), the uNK cell enrichment in the conceptus attachment sites of pigs is not pronounced, but only reaches about 3-fold. The immunological response at the maternal-fetal interface especially in mice and humans are characterized by intrauterine immune suppression, non-classical MHC molecule expression by trophectoderm and immune-protective molecules including Fas ligand (CD95) expression by the trophoblast (66–68). The porcine placental cells do not express MHC class I molecules (swine leukocyte antigens [SLAs]) (69), however, the stromal cells and luminal epithelial cells express classical and non-classical SLA class I molecules (SLA-1,−2,−3,−6,−7, and−8) as well as β2-microglobulin (70). Primarily involved in antigen presentation, MHC class II molecules (SLA DQA) demonstrate a greater expression in subepithelial stromal cells and endothelial cells of the endometrium in response to conceptus-derived IFN-γ during the window of conceptus attachment (71). The conceptus likely modulates SLA-DQA expression and hence indirectly influences the maternal immune system for its survival.

## microRNAs Involved in Regulation of Angiogenesis and Immune Cell Development

The regulation of gene expression at the porcine maternal-fetal interface is controlled by several biomolecules at the genomic level. One of the most important implicated biomolecules are microRNAs (miRNAs), a class of small non-coding RNAs (~18–22 nucleotide in length) that play a major role in RNA silencing and post-transcriptional gene regulation (72). MiRNAs negatively regulate translation either by deadenylating and destabilizing the mRNA or by repressing the translational machinery. miRNAs are primarily involved in regulating both physiological and pathological processes including but not limited to cellular proliferation and differentiation, homeostasis, inflammatory processes, and cell death (72, 73). Most mammalian miRNAs are highly conserved among species (74, 75) and regulate at least 70% of all the genes in their specific genome (76). Each miRNA can repress the expression of multiple target mRNA, while each mRNA target is regulated by many miRNAs, suggesting that the regulation of gene expression is highly complex.

Because of their unique expression pattern in both physiological and pathological processes, miRNAs are projected as biomarkers of both healthy pregnancy and associated disorders. In one study, a set of 17 miRNAs were identified in the porcine placenta samples obtained from pigs at gd 30 and 90. Differentially expressed 8 miRNAs between the two time points, such as let-7i, miR-106a, miR-17, miR-24, miR-92b, miR-125b, miR-20, and miR-27a were validated by stem-loop RT-PCR. Further bioinformatic analysis of mRNA targets and potential biological pathways indicated that these miRNAs are involved in regulation of cell growth, trophoblast differentiation, maintenance of adherence junctions and angiogenesis (77). miRNAs appear to follow a pattern of expression depending on the overall dynamics of cellular processes, that are typical of porcine pregnancy. Based on the temporal expression patterns, a study had identified 65 miRNAs in the endometrium to capture crucial stages of pregnancy such as conceptus attachment (gd 15), placental development (gd 26), and mid-gestation (gd 50) (78). These miRNAs appear to regulate the processes associated with embryo attachment, placental development and overall homeostatic mechanisms associated with pregnancy. This study further characterized the binding sites of two miRNAs, miR-181a and miR-181c in the transcripts of genes such as ITGB3, ESR1, and SPP1, which are proven to play a crucial role in embryo implantation (78).

Studies over the past decade have characterized several miRNAs that regulate various porcine reproductive and pregnancy associated processes, including oocyte and conceptus development (35, 79, 80), implantation (35, 78), immune cell recruitment, and placental angiogenesis (29, 33), placental growth and function (77), and litter size (81–83). Specific examples include miRNA-29a that triggers the degradation of basement membrane and stromal matrix by interacting with *LAMC1* (encodes laminin subunit gamma 1), *COL1A2* (encodes collagen type 1 alpha, chain 2), and *COL3A1* (encodes collagen type 3 alpha, chain 1). Further, miR-200 family and miR-205 shown to control the trophoblast epithelial cell adherens junctions by interacting with *ZEB2* and *CDH1*. Similarly, miR-17-92 regulate trophoblast proliferation by interacting with *HBP1* and *ULK1* miRNAs (84). The miRNA synthesis and their transportation across the cellular compartments is also a highly regulated process. A set of 10 genes namely *DGCR8, AGO3, AGO4, XPO5, AGO1, AGO2, DICER1, DROSHA, TNRC6A*, and *TARBP2* that code for proteins involved in miRNA synthesis and transport have been investigated for their role in the endometrium of pigs. Using bioinformatics approach, this study identified target transcripts, VEGF, IL-6R, LIF, and PTGS2, that play a crucial role in maternal-fetal communication (35). Similarly, a distinct set of genes that regulate miRNA synthesis and transport (*DGCR8, TNRC6A, DROSHA, XPO5, DICER, AGO1 to*−*4*, and *TARBP2*) as well as miRNAs such as miR-140 were investigated in pregnant and cyclic endometria obtained on gd 12, 16, and 20 in pigs. The expression of *DROSHA, XPO5, DICER1, TARBP*, and *AGO1* was altered by the reproductive status of animals. Overall, these studies suggest that the interaction between miRNAs and genes involved in miRNA synthesis and transport likely involved in the regulation of estrous cycle and initial stages of embryo attachment (79).

Studies from our group evaluated the expression of 236 miRNAs in endometrial specimens and fetal trophoblasts derived from both the healthy and growth arrested conceptus attachment sites at gd 20. We reported significantly higher expression of miR-331-5p, 330-5p, 323, and 935, while miR-10a, 27a, 29c, and 374b-5p in the endometrial samples associated with healthy attachment site. These were one of the initial miRNA array-based studies to highlight the importance of miRNAs in the porcine pregnancy. Importantly, in this study, we found that the differentially expressed mRNAs indeed regulate many biological processes associated with pregnancy such as blood vessel development, nuclear transcription factor regulation, and extracellular matrix factors (85).

Due to the non-invasive superficial attachment, the epitheliochorial placentation in pigs enables us to easily isolate both maternal and fetal tissues to study molecular mechanisms without intermixing the compartments. Angiogenesis is one of the major mechanisms determines the success of implantation and growth of fetus. Therefore, our initial studies on immune-angiogenesis axis prompted us to explore miRNA regulation in recruited immune cells to the endometrium during pregnancy. Using laser capture microdissection, we isolated lymphocytes from the endometrium associated with both the arresting and healthy conceptus attachment sites and conducted targeted miRNA profiling on these immune cell subsets. Our major focus was to study miRNAs that are reported to be associated with immune cell development and overall pregnancy associated functions including angiogenesis. Interestingly, a distinct set of miRNAs such as miR-17P-5P, miR-18a, miR-19a, miR-150, and miR-296-5P were differentially expressed in the endometrial lymphocytes obtained from healthy conceptus attachment sites compared to their arresting counterparts. Further, there was significant differences in the expression of miR-17-5P, miR-20b, and miR-18a in the endometrium isolated during early pregnancy stages at gd20 (29). These studies provide concrete evidence into the role of miRNAs in the immune cell differentiation in the porcine endometrium and their expression levels changes depending on the pregnancy status. More importantly, this study also demonstrates that angiogenic miRNAs are enriched in the endometrial lymphocytes and can regulate immune cell promoted angiogenesis at the maternal-fetal interface to support developing conceptus (29).

## Biogenesis and Secretory Mechanism of Extracellular Vesicles

Extracellular vesicles (EVs) is a general term used to describe membrane bound vesicles secreted by several cells for differing functions. Although previously thought to be exclusively intended for the secretion and elimination of waste, EVs contain coding and non-coding nucleic acids, lipids, and proteins, which are highly relevant for intercellular communication, homeostatic processes and also contribute to pathologies including those at the maternal fetal interface (34, 86, 87). Although heterogeneric in size, composition, origin, and function, EVs are generally categorized into two groups: exosomes and microvesicles (MV). Exosomes range in size from 30 to 150 nm while MV range from 150 nm to 1 μm in diameter. In addition to size, morphology [described as round or saucer shaped (88)] and specific markers are used for categorization. Due to significant controversy surrounding the proper categorization of EVs together with a rise in scientific publications in recent years, the International Society for Extracellular Vesicles have proposed specific guidelines and protocols to direct the nomenclature, isolation of pure EV preparations and proper categorization of EVs (89). Briefly, isolation techniques of EVs include differential ultracentrifugation, density gradient centrifugation, filtration, and size exclusion chromatography. Characterization to confirm size, morphology, and marker presence is conducted by immunoblotting, flow cytometry, transmission electron microscopy, or nanoparticle tracking analysis. Although one marker does not exclusively identify EVs, proteins such as tetraspanins (including CD9, CD63, and CD82), as well as other sorting complex proteins are used to identify EVs (in both exosomes and MV).

Biogenesis and secretion of exosomes compared to MV is different. MV are released *via* budding of the plasma membrane and are thought to be released faster, as the cargoes targeted by these are situated at the plasma membrane. Whereas, exosomes are first formed as intralumenal vesicles (ILVs) within multivesicular endosomes (MVEs) of the cell and secretion requires sorting of cargoes through endosomal-sorting-complex-required-for-transport (ESCRT) dependent or independent pathways. Recent studies in the field have explored the possibility of sorting machineries, especially ESCRT complexes to be involved in decision making to target MVEs for either degradation or secretory pathway (90) through ALIX (ESCRT III associated complex) dependent mechanism (91). Similarly, another ESCRT complex associated protein (TSG101) has been studied by Villarroya-Beltri et al., where, a key posttranslational ubiquitin-like modification, ISGylation of TSG101 has been identified to be the probable signaling mechanisms that drives MVEs to fuse with lysosomes to induce degradation instead of secretion (92). Regardless of the outcomes, MVEs need to be transported continuously to either fuse with lysosomes or plasma membrane through general cellular transportation molecules such as actin, microtubule cytoskeleton, cortactin, RAB protein complexes and their effector molecules (93, 94). Studies in cancer cell lines have shown that actin cytoskeleton to be involved in the transportation and secretion of exosomes (94). Transportation of vesicles within the cells, including budding and fission of MVEs at the plasma membrane is tightly governed by GTPases-RAB protein family that consists of up to 60 members.

Even though the exact mechanism of action is not understood, members of RAB protein family such as RAB2B, RAB4, RAB5A, RAB7, RAB9A, RAB11, RAB27A, RAB27B, and RAB35 have been found to be involved specifically in endosome transport to plasma membrane and secretion of exosomes, as discussed elsewhere (95). Finally, fusion of MVEs to the plasma membrane is necessary to release ILVs as exosomes, which is thought to be primarily mediated by N-ethylmaleimide-sensitive factor attachment protein receptors (SNAREs). SNARE protein complexes are made up of three to four subunits forming helices that are conventionally involved in exocytosis of lysosomes (96, 97). Recent developments in the field has identified a few proteins of SNARE family members namely, vesicle-associated membrane protein 7 (VAMP7), Synaptobrevin Homolog YKT6, syntaxin 1A and syntaxin 5 to be crucial to facilitate the release of exosomes. Depending on the subtypes of MVEs, organism and cell type, diverse regulation of secretion by SNARE proteins have been identified in human embryonic kidney HEK293 cells (98), lung carcinoma A549 cells (99), *Drosophila* (98) (YKT6), and C. *elegnas* (syntaxin 1a and syntaxin 5) (100, 101). However, the exact stage where these molecules are recruited is yet to be identified. This limitation is mainly regarded to the technical approach as well as intracellular complexity of exosome release.

Interestingly, the release of EVs is an evolutionarily conserved process in both prokaryotes and eukaryotes and therefore EVs serve diverse biological functions, reviewed extensively here (102). As mentioned earlier, EVs including exosomes contain lipids, nucleic acids (mRNA, miRNA, DNA), enzymes, transcription factors and extracellular matrix proteins packaged by parental cells that exert drastic effects in the recipient cell (103). Conventional cell to cell communication occurs through surface protein interaction with neighboring cells, whereas hormones and cytokines are recruited to fulfill distant interactions (104). Considering the contents of exosome cargo, number of studies have centralized the approach toward understanding the molecular interactions between exosomes and recipient cells. Although the entire mechanism is not fully understood, it is known that exosomes are required to fuse with the plasma membrane, followed by signal transduction and internalization (endocytosis, phagocytosis, and micropinocytosis) with the target cells in order to deliver the cargo and establish intercellular communications. Known mechanism of action includes interaction with surface bound receptors, epigenetic modification *via* genetic material and activated receptor transfer to the target cell. Transmembrane proteins (integrins, tetraspanins) and extracellular matrices are primarily involved in binding exosomes to the target cells. Adhesion proteins such as intracellular adhesion molecules (ICAM) have been identified to interact during uptake with the integrins present on exosomes (105). Specific route of internalization is thought to be influenced by the content of molecules carried by exosomes. Studies in human pancreatic cancer cells have identified mutant KRAS gene to influence the exosomes to be internalized *via* micropinocytosis (106). Similarly, complexity of exosome action surrounding specific cell targeting is believed to be determined based on the cargo carried by exosomes.

## Extracellular Vesicles in Porcine Pregnancy

**Figure 1:**
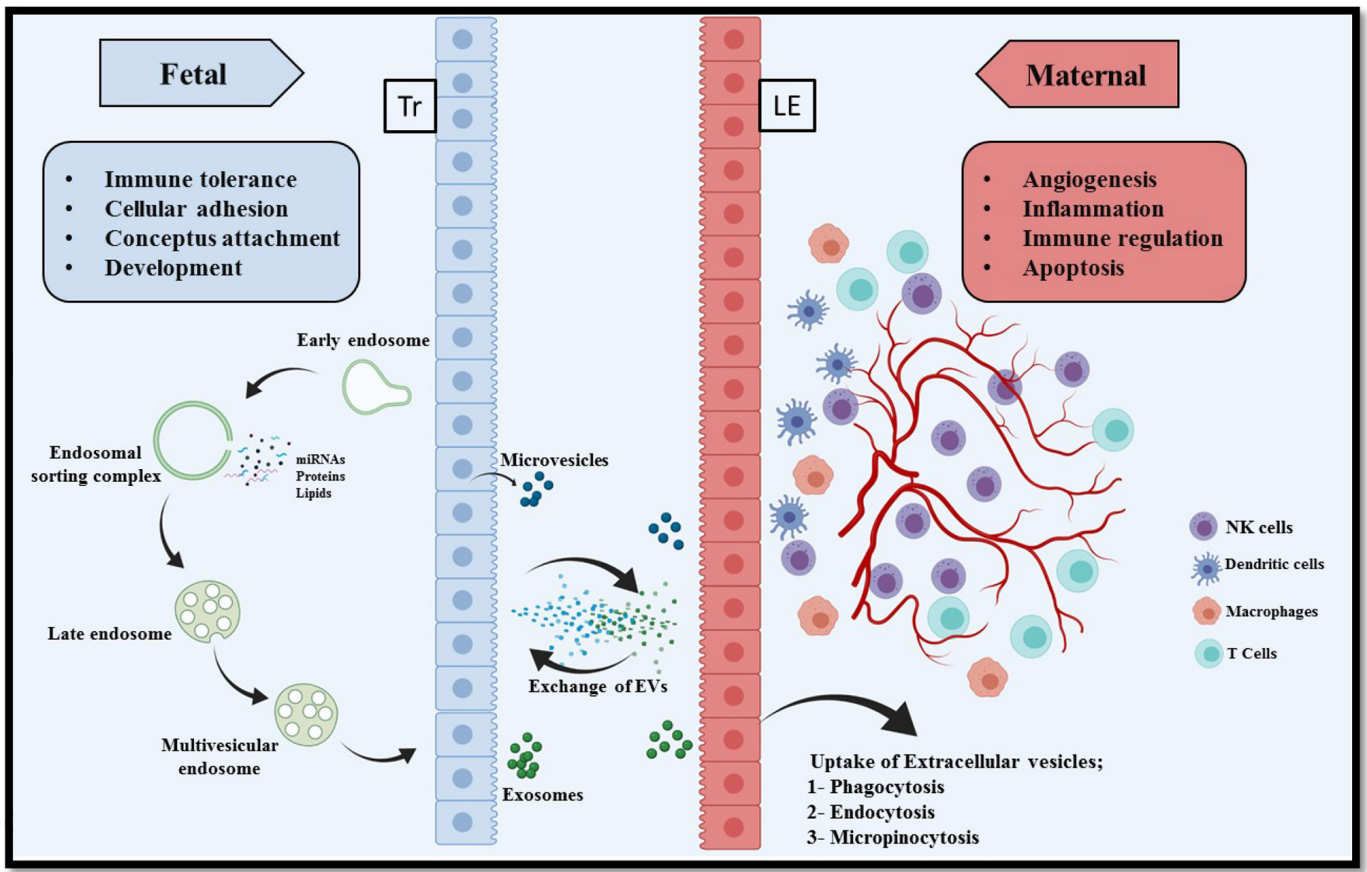
Extracellular vesicle mediated bi-directional communication at the porcine maternal fetal interface. In epitheliochorial placentation in pigs, fetal trophoblasts (Tr) lay in simple apposition with maternal endometrial luminal epithelium (LE). Extracellular vesicles comprising microvesicles and exosomes are a heterogeneous group of cell-derived membranous structures originated from either endosomal system (microvesicles) or shed from the plasma membranes (exosomes). The unique cargo of these EVs contain biomolecules (nucleic acid, proteins, and lipids). These EVs can be internalized by multiple pathways including phagocytosis, endocytosis, pinocytosis, or can remain bound to the cell surface depending on the cell type. Interaction of EVs with recipient cells modulate several critical pregnancy associated processes, for example, conceptus attachment, development, immune tolerance on the fetal side and angiogenesis, inflammation, and apoptosis on the maternal side.

A successful conceptus attachment to the endometrium and subsequent maintenance of pregnancy requires a proper communication between endometrium and conceptus (11). There are already several well-understood mechanisms of cell-cell communication such as hormones, cytokines, and lipids that act in an autocrine and paracrine manner, intercellular nanotubules (107), direct adhesion between cell of origin and target cell, and membrane-bound microvesicles (108). Research over the past decade has provided critical evidence into intercellular communication *via* EVs (109). The EVs have been demonstrated to transfer information from the cell of origin to the recipient cells to regulate cellular activities (Figure 1). This information could be in the form of proteins, lipids, miRNAs, mRNAs, DNA and many other small molecules, and thus reflecting the physiological state of the originating cells (110, 111). However, the transfer of information could be two way, where the recipient cell can in turn stimulate/induce the originating cells in order to elicit the cellular signaling pathways (112, 113).

The bioactivity possessed by these EVs is distinct. Therefore, EVs have the ability to influence both physiological (110) and pathological processes (111). However, the precise mechanisms regulated by exosomes is difficult to understand, due to their complexity of cargo, abundance, cell of origin, biogenesis and secretory mechanisms, and extracellular environment as discussed in earlier section. Another important characteristic of the exosomes is their stability in the extracellular spaces due to their lipid bilayer containing cholesterol, ceramide, sphingomyelins and other detergent-resistant membrane molecules, which are likely derived from the originating cells (114). These unique features allow them to carry information without leaking into the extracellular spaces. Upon entering the recipient cell, the exosomes dissociate to release cargo, which may regulate gene expression and influence biological processes such as cell proliferation, differentiation, and migration. The exosomes have been demonstrated to be released by cells of the reproductive tissues such as endometrial (115), oviductal (116), and follicular cells (117) as well as bodily fluids such as saliva (118), blood plasma (119), milk (120), amniotic fluid (121), semen (122), urine (123), and uterine luminal fluid (35). The exosomes carried through different bodily fluids can selectively interact with target cells and deliver a diverse set of molecules to influence target cell activity suggesting that exosomes provide a highly regulated and complex mode of cellular communication.

Although there are many mechanisms of communication between conceptus and maternal endometrium, the documentary evidence is increasing exponentially about EVs/exosomes being the important mode of communication at the maternal-fetal interface. However, this is not a totally new concept as the discovery of small vesicles goes back to early 1990's, where multiple small vesicles have been shown at the porcine maternal-fetal interface on gd16 *via* electron microscopy (12). Research in recent years have shown that EVs including exosomes released by placental cells can cross into maternal endometrium to deliver cargo containing biomolecules to the recipient cells.

It already known that the exosomes have the ability to transfer many important biomolecules such as mRNA and miRNAs to their respective target cells, in which mRNA can be directed toward translating into protein and miRNA can be utilized to fine tune mRNA expression or repress translation (124, 125). Exosomes carrying these biomolecules can attach to the surface of target cell (126) and can be internalized by recipient cells (127, 128). Chromosome 19 miRNA cluster generates more than 50 miRNAs and this cluster appear to modulate immune response in pregnancy. The immunomodulatory mechanism is essential especially in early pregnancy to help recently attached conceptus to grow and favorably modulate maternal immune response to paternal antigen. Interestingly, one study demonstrated a viral resistance in recipient cells that received miRNAs that were packaged within the human trophoblast-derived exosomes (129).

Another interesting concept is that MV and exosomes can be released in a time dependent manner as well as based on the physiological status. For example, the secretome released from placenta during first trimester of human pregnancy predominantly consisted of exosomes (130), while during the second trimester, the composition of the secretome changed predominantly to microvesicles and also their number increased as the pregnancy advanced to term (131, 132). Similarly, the function regulated by these ever-changing components of microvesicles and exosomes also differs greatly depending on the stage of pregnancy. For example, the exosomes released during early human pregnancy contain HLA-G (MHC I G), that help in enabling maternal immune tolerance toward recently attached conceptus carrying paternal antigens (130). Furthermore, MV released by syncytiotrophoblasts induce pro-inflammatory activity, which is essential around the stages of pregnancy establishment (133). However, these dynamic variations in the components of secretome need to be investigated in pigs to understand how trophoblast derived EVs modulate maternal adaptions to pregnancy given the non-invasive type of placentation.

EVs have been shown to perform a variety of pregnancy associated functions that most often reflect the constituents of its cargo. Conceptus attachment sites in pigs undergo dramatic changes both during successful and abortive pregnancy. For example, conceptus attachment sites associated with arresting embryo undergo an acute inflammatory process to enable resorption without affecting the adjacent littermate. Apoptosis can also get elicited in the conceptuses to remove or replenish the unnecessary cells. This mechanism of programmed cell death has been reported in many reproductive tissues such as uterine epithelium (134). Human placenta derived exosomes have been shown to carry Fas ligand and TRAIL molecules and induce apoptosis in activated immune cells and thus providing maternal immune tolerance toward developing fetus (135). Similarly, a regulatory protein such as bcl-2-like protein 15 (BCL2L15) has been reported in ovine uterine EVs during the periattachment period, gd17 (136). Another study in cows demonstrated that EVs obtained from gd17 induced greater expression of apoptotic-related genes, BAX, TNFA, TP53, and CASP3 in primary endometrial epithelial cells (137). These findings indicate a crucial role for EVs in apoptotic removal of immune cells (immunomodulation) and endometrial epithelial cells to help in uterine remodeling for conceptus attachment.

A recent study has isolated and characterized the EVs in porcine uterine flushing fluid collected on gd10, 13, and 18. Contents of these EVs especially small RNAs were comprehensively profiled through small RNA sequencing analysis. The cargo consisted of 152 known miRNAs, 43 novel miRNAs, 6248 known Piwi-interacting RNAs (piRNAs), and 110 novel piRNAs were identified. Subsequent bioinformatics analysis revealed that the miRNA enriched in the EVs involved in important pregnancy associated pathways such as immunomodulation, endometrial receptivity, implantation and embryo development. These studies add to the miRNA repository as well as serve as a resource to further investigate crosstalk at the maternal-fetal interface (138). As integrin family proteins play an important role in embryo implantation, previous study demonstrated that EVs in the bovine uterine flushings isolated on gd20 and 22 were able to upregulate VCAM1 expression in endometrial epithelial cells (139). Similarly, a study involving exosomes from estrogen or progesterone treated human endometrial epithelial cells contained several members of integrin family. The integrins are crucial for exosome docking to target cells and regulate trophoblast adhesion to the endometrium (140). EVs isolated from uterine lumen flushings on gd12, 14, and 16 reported to contain miR-26a and miR-125b and among these miRNAs, miR-125b directly regulated expression of genes that play role in attachment and embryo development in pigs (35). These studies support the notion that EVs can influence conceptus attachment and adhesion to the endometrium. However, given fundamental differences in the placentation between human, mice and pigs, a pig centric studies are warranted to investigate mechanisms by which the biomolecules packaged in EVS/exosomes regulate porcine pregnancy related functions.

Placental angiogenesis is an important pregnancy related processes crucial for conceptus development and remodeling of endometrium especially in early pregnancy to provide nutrients to the fetus. The placentation in pig typically initiates gd 15–20, which involves an abrupt change in endometrial microenvironment and associated biological processes including angiogenesis (141, 142). Studies from our laboratory have investigated angiogenesis at the porcine maternal-fetal interface and recently, we demonstrated that both the porcine trophectoderm cells, representing fetal tissues and endothelial cells, representing maternal tissues release EVs (34). Furthermore, these experiments demonstrate that EVs derived from porcine trophectoderm cells can stimulate endothelial cell proliferation, suggesting that EVs can stimulate angiogenesis. We have shown that EVs derived from porcine trophoectoderm cells (PTR2) contains several important proteins and miRNAs such as miR-126-5P, miR-296-5P, miR-16, and miR-17-5P that have been shown to play a major role in angiogenesis (34). miR-150 packaged within exosomes derived from porcine umbilical cord blood stimulated proliferation, migration, and tube formation of umbilical vein endothelial cells. These studies indicate that EVs especially those derived from fetal tissues have the ability to stimulate endometrial angiogenesis and thus, support the idea that an angiogenic deficit at the maternal-fetal interface can be therapeutically targeted by designer EVs and prevent subsequent conceptus loss.

The development of immunological tolerance against semi-allogeneic fetus is a crucial event in the pregnancy success. Since fetal allografts carry paternal antigens, the maternal immune system should reprogram itself to not to recognize fetal antigens and elicit an immune response. EVs have been reported to modulate maternal immune response toward the newly attaching conceptus (143). Depending on the nature of cargo and also type of receptors present on their membrane, EVs are able to modulate the immune response at the maternal-fetal interface. Bovine EVs obtained from gd20, were able to downregulate expression of immune-related genes in endometrial epithelial cells immediately after the conceptus attachment is initiated (144). Zhao et al. reported in bovine pregnancy that bta-miR-98 as a likely maternal immune system regulator based on the miRNA profiles of EVs combined with bioinformatic analysis. Similarly, miRNA-499 was reported to play a role in regulation of local inflammation at the bovine maternal-fetal interface by inhibiting NF-kB signaling. Further, disruption of miR-499 results in increased risk of pregnancy failure due to severe local inflammatory process, placental resorption in early pregnancy or even fetal growth restriction (87). These studies suggest that miRNA cargo present in the EVs can modulate immune response, ultimately, helping to achieve immune tolerance during early pregnancy. Although, our previous studies in pigs have pointed out a distinct set of miRNAs that may have immunomodulatory effect on the maternal immune system, more mechanistic *in vivo* evidence is needed to exploit the idea that miRNA loaded EVs can be used to potentially reduce spontaneous fetal loss in pigs in future.

## Extracellular Vesicles in Selective Reproductive Disorders in Pigs

Extracellular vesicles in porcine pregnancy: Early pregnancy diagnosis or detecting early embryonic mortality could substantially improve reproductive efficiency in pigs and overall improve the economy of swine industry. Failure to conceive after insemination or undetected pregnancy at an early stage in pigs could result in severe economic loss. While there are many well-established techniques currently available, miRNAs and miRNAs packaged within the exosomes could be another important tool to be used as the earliest possible (before 25 days after fertilization) biomarkers of pregnancy (145). Zhou and colleagues profiled exosomes obtained from serum samples at days 9, 12, 15 from pregnant and non-pregnant pigs. They concluded that miR-92b-3p and miR-17-5p could be identified in the serum exosomes as earlier as day 9 of pregnancy and hence could be used as biomarkers of early pregnancy (145).

Interestingly, in one of our previous studies miR-17-5p was one of highly enriched miRNA present in the porcine trophectoderm cells, derived from day 12 of pregnancy, and porcine endothelial cells as well as exosomes released by these two cell types (34). miR-17-5p is involved in regulating many physiological and pathological processes including cell proliferation, apoptosis, and most importantly angiogenesis. In addition, the genes targeted by miR-17-5p are reported to be involved in important signaling pathways including MAPK, PI3K-Akt, and TGF-β. Similarly, a study involving cattle identified 27 circulating EV-derived miRNAs isolated from serum that were significantly increased on gd 17 embryonic mortality compared to pregnant cattle. Furthermore, a specific miRNAs such as miR-25, miR-16b, and miR3596 were differentially expressed on gestation days 17 and 24, reflecting the pregnancy status (146). In another study, a set of 27 miRNAs packaged within EVs from maternal blood were found to be in lower abundance in somatic cell nuclear transfer-derived bovine embryonic loss group that failed to reach term. Additionally, the predicted target genes of these 27 miRNAs were found to be associated with critical biological processes such as cell proliferation, apoptosis, and angiogenesis (147).

Role of extracellular vesicles in porcine reproductive and respiratory syndrome pathogenesis: EVs have been shown to play a role in diseases affecting reproductive organs in pigs. Porcine reproductive and respiratory syndrome (PRRS) is one of the important diseases affecting reproductive organs in pigs and causes a heavy economic loss to pork industry. It is characterized by reproductive failures in sows and respiratory syndrome in pigs of all ages (148). An emerging evidence indicates that exosomes released by cells infected by some viruses selectively package viral genetic material, viral proteins, or virions to transmit to neighboring healthy/uninfected cells (149). Purified exosomes isolated from PRRS virus infected cells contain viral genomic RNA and partial viral proteins. These exosomes loaded with viral components can deliver their contents to both PRRS virus susceptible and non-susceptible cells, indicating a viral transmission *via* exosomes while evading the host immune response (150).

Exosomes can serve as small RNA transfer vehicles and act as effective therapeutic tools. Zhu and colleagues conducted a study to target two key receptors, Sialoadhesin (Sn) and CD163 for PRRS virus infection of porcine alveolar macrophages. They designed artificial miRNAs that can directly target Sn and CD163 as PRRS virus enters the target cells *via* receptor-mediated endocytosis. They generated two recombinant adenoviruses expressing the effective artificial miRNAs. It was observed that sequence-specific artificial miRNAs were expressed adequately and released from recombinant adenovirus transduced pig cells *via* exosomes. Overall, PRRSV infection of pulmonary alveolar macrophages was inhibited by transduction of two artificial miRNA expressing recombinant adenoviruses and or treatment with two artificial miRNAs packaged within the exosomes. This study suggest that exosomes can be utilized to effectively treat infectious diseases such as PRRS (151). Another study by the same group, used exosome mediated transfer of artificial miRNA targeting the 3' untranslated region of PRRSV. They generated a recombinant adenovirus expressing the artificial miRNA that can target 3' untranslated region of PRRSV and further concluded that exosomes derived from porcine cells can be utilized as miRNA cargo and miRNAs delivered *via* exosomes effectively elicited anti-viral effects against different PRRSV strains (152). Montaner-Tarbes et al. isolated serum-derived exosomes from naïve animals, from PRRSV viremic animals and from animals recovered from PRRSV infection and already free of viruses (non-viremic). Their experiments suggest that the serum-derived exosomes contain antigenic viral-proteins, which could be used as a novel vaccine strategy against PRRSV infection (149). Further this group conducted a targeted-pig trial on safety and immunogenicity of serum derived EVs containing viral proteins and concluded that this could be an effective vaccine strategy (153).

## Summary and Conclusion

Co-ordinated interactions between maternal endometrium and fetal trophoblasts at the highly dynamic interface is critical to ensure pregnancy success. In epitheliochorial placentation seen in pigs, the fetal trophoblasts lie in simple opposition with luminal epithelial cells of the endometrium but there is no invasion. The significant alterations in the uterine stroma as seen in other species such as human and mice is also lacking in pigs except localized branching of new blood vessels to support the needs of a growing conceptus. We and others have shown that the conceptus mediates the recruitment of immune cells that adopt a specialized phenotype and these immune cells contribute to and regulate angiogenesis at the porcine maternal-fetal interface. It is important to recognize that because of the non-invasive placentation in pigs, trophoblasts do not physically interact with maternal-immune cells. In fact, trophoblasts in contact with luminal epithelium lacks SLA class I and II molecules and therefore avoid recognition by the maternal-immune cells. Maternal-immune cells predominantly NK cells and T cells are recruited around gd-12-15 at the maternal-fetal interface by conceptus mediated signals as well as specific sets of chemokines. The pro-inflammatory microenvironment and chemokine gradient is regulated by chemokine decoy receptors such as D6 and DARC. Recent evidence indicate that immune cell differentiation and function is also regulated by specific sets of miRNAs. Indeed, miRNAs are involved in modulating several physiological, homeostatic, and pathological processes including vascularization and inflammation. Several reports also indicate suitability of unique miRNA signatures in body fluids as predictors of health and disease.

The paradox of bi-directional communication between maternal and fetal compartments can be better explained by extracellular vesicle mediated inter-cellular cross talk, especially in species with epitheliochorial placentation where physical interactions between trophoblast and maternal immune cells is limited. The EV cargo containing genomic DNA fragments, RNAs, mRNAs, miRNAs, proteins, and lipids are specifically packaged and can be traced back to the cell of origin. The EV cargo contents are also influenced by the localized microenvironment cues and are involved in embryo implantation, placentation, pregnancy maintenance, and pregnancy associated disorders. With the advent of newer sequencing technologies and bioinformatics machine learning tools, the contents of EVs and their utility in understanding physiological changes in pregnancy and spontaneous fetal loss will be of immense value in future.

## Author Contributions

MB, MK, and CT conceived ideas and associated themes. HL and JM contributed sections of the manuscript. MB, HL, JM, MK, and CT wrote and edited manuscript. CT provided research funding and oversight. All authors contributed to the article and approved the submitted version.

## Conflict of Interest

The authors declare that the research was conducted in the absence of any commercial or financial relationships that could be construed as a potential conflict of interest.
